# Associations of Perceived Stress and Social Support on Health Behavior Changes in Sexual Minoritized Women During the COVID-19 Pandemic

**DOI:** 10.1089/whr.2022.0095

**Published:** 2023-04-17

**Authors:** Donghee N. Lee, Elise M. Stevens, Joanne G. Patterson, Amelia V. Wedel, Theodore L. Wagener, Brittney Keller-Hamilton

**Affiliations:** ^1^ Division of Preventive and Behavioral Medicine, Department of Population and Quantitative Health Sciences, University of Massachusetts Chan Medical School, Worcester, Massachusetts, USA.; ^2^ Division of Health Behavior and Health Promotion, College of Public Health, The Ohio State University, The Ohio State University Comprehensive Cancer Center, Columbus, Ohio, USA.; ^3^ Center for Tobacco Research, The Ohio State University Comprehensive Cancer Center, Columbus, Ohio, USA.; ^4^ Department of Psychology, Syracuse University, Syracuse, New York, USA.; ^5^ Division of Medical Oncology, Department of Internal Medicine, The Ohio State University College of Medicine, Columbus, Ohio, USA.

**Keywords:** COVID-19, LGBTQ, sexual minority women, perceived stress, social support, health behavior

## Abstract

**Purpose:**

We examined how perceived stress and social support were associated with changes in health behaviors during the COVID-19 pandemic among sexual minoritized women (SMW).

**Methods:**

In an online convenience sample of SMW (*N* = 501, *M*_age_ = 23.6), we used multinomial logistic regression models to estimate associations of perceived stress and social support (emotional, material, virtual, in-person) with self-reported changes (increased or decreased vs. no change) in fruit and vegetable intake, physical activity, sleep, tobacco, alcohol, and substance use during the pandemic. We also tested whether social support modified associations between perceived stress and changes in health behaviors. Models controlled for sexual orientation, age, race and ethnicity, and income.

**Results:**

Perceived stress and social support were associated with changes in health and risk behaviors. Specifically, increased perceived stress was associated with decrease (odds ratio [OR] = 1.20, *p* = 0.01) and increase (OR = 1.12, *p* = 0.04) in fruit and vegetable intake, and increase in substance use (OR = 1.19, *p* = 0.04). Receiving in-person social support was associated with changes in decrease (OR = 10.10, *p* < 0.001) and increase (OR = 7.35, *p* < 0.01) in combustible tobacco use and increase in alcohol use (OR = 2.63, *p* = 0.01). Among SMW who never received material social support during the pandemic, increased perceived stress was associated with increased alcohol use (OR = 1.25, *p* < 0.01).

**Conclusions:**

Perceived stress and social support were associated with SMW's health behavior changes during the pandemic. Future research may explore interventions to mitigate the effects of perceived stress and appropriately increase social support to promote health equity among SMW.

## Introduction

The COVID-19 pandemic has disproportionately impacted the health of the lesbian, gay, bisexual, transgender, and queer (LGBTQ) community.^1–3^ Forty percent of LGBTQ individuals (vs. 22% of heterosexuals) are essential workers serving in the hospitality, medical, education, and retail industries, which increases their risk of COVID-19 exposure.^4^ LGBTQ individuals have experienced increased economic sensitivity compared with their heterosexual counterparts, including unemployment (14% vs. 12%, respectively), reduced work hours (30% vs. 22%, respectively), poverty (22% vs. 16%, respectively), greater financial concerns (20% vs. 11%, respectively),^2^ and lack of access to health insurance (17% vs. 12%, respectively),^4^ which could increase their stress and affect their ability to cope with COVID-19. This socioeconomic disparity is especially concerning, as LGBTQ adults already experience health disparities.^5–11^

Within the LGBTQ community, sexual minoritized women (SMW) (*i.e.*, individuals who self-identify as female or were assigned female sex-at-birth, and who experience oppression due to their non-heterosexual identity; SMW) might be particularly vulnerable to the negative impact of COVID-19. Compared with heterosexual women and sexual minoritized men, SMW display disparately high prevalence of tobacco, alcohol, and substance use.^5–8^

According to the minority stress model, such risk behaviors reflect a mechanism to cope with excess stress arising from marginalization and discrimination due to their minoritized sexual orientation.^9–14^ The SMW's disparate exposure to these stressors has increased during the COVID-19 pandemic, as SMW have reported greater levels of COVID-19 related stress compared with heterosexual women^15–17^ and increased harassment, involuntary disclosure of sexual identity, and discrimination compared with pre-pandemic.^15,18^ Despite these disparities, little attention has been paid to examine how stress may explain changes in SMW's health and risk behaviors.

Per the minority stress model,^19^ SMW who receive social support may be less likely to engage in health risk behaviors, and certain types of social support may moderate the effect of stress on health behaviors among SMW. For example, receiving in-person support is associated with reduced risk of tobacco use disorder comorbidities^20^ and being able to talk openly about their sexual orientation and gender identity in a supportive setting is associated with increased likelihood of tobacco non-use.^21^

Moreover, social isolation is associated with increased use of alcohol, tobacco, and cannabis.^22^ Emerging evidence also indicates that receiving social support was associated with increased resilience and positive coping among LGBTQ people during the pandemic.^23–26^ However, increased number of friends from LGBTQ networks has also been associated with greater tobacco and alcohol use,^27^ possibly due to cultural norms that support using substances to cope with identity-specific stress.^28,29^ As such, different types of social support may promote or impede health and risk behaviors among SMW.

During the COVID-19 pandemic, individuals' access to social support changed due to the implementation of preventive measures to contain the spread of COVID-19, including lockdowns, social distancing, and the closing of mental health programs^30^ and LGBTQ-specific support groups.^31,32^ But research has yet to examine how social support may promote or impede SMW's changes in health and risk behaviors during the COVID-19 pandemic.

It is possible that increased stress and decreased social support might contribute to the poorer health indicators and increased prevalence of risk behaviors evidenced among SMW during the COVID-19 pandemic. Compared with their heterosexual counterparts, SMW have demonstrated poorer health behaviors, including worse nutritional quality, overeating, and stress eating from the beginning of COVID-19.^33,34^ Substance use behaviors have also disproportionately increased among SMW, including increased use of cannabis and alcohol to cope with COVID-19 related depression and anxiety.^33,35,36^

Decreased social support during the COVID-19 pandemic has further contributed to increased loneliness, depression,^31,32,35^ and alcohol use.^35^ Despite this problem, limited evidence has evaluated how perceived stress and social support are associated with changes in health and risk behaviors among SMW during the COVID-19 pandemic.

### The current study

The goal of the current study was to empirically examine how perceived stress and social support types (emotional, financial or other in-kind material, such as money) and platforms (virtual, in-person) were associated with health and risk behavior changes during the COVID-19 pandemic among young SMW. Overall, we hypothesized that greater perceived stress would be associated with lower odds of engaging in healthy behaviors and higher odds of engaging in risk behaviors, whereas greater social support would be associated with higher odds of engaging in healthy behaviors and lower odds of engaging in risk behaviors. In addition, we hypothesized that social support would moderate the association between perceived stress and health and risk behaviors among young SMW.

## Methods

### Setting

In January 2021, we used convenience sampling to recruit participants into a survey in Prolific, an international crowdsourcing platform for behavioral research studies. Prolific is a strong platform for conducting scientific research because of its transparency and high functionality.^37^ We implemented several measures to ensure quality of data. For example, participants were provided with a compensation code at the end of the survey and asked to answer open-ended questions to identify “bot” or automated responses. In addition, collected data were manually reviewed to identify duplicate or fraudulent responses.

### Sample

Participants (*N* = 501) were eligible to participate in the study if they were between 18 and 30 years old; self-identified as young adult women or assigned female-at-birth and identified as lesbian, bisexual, or another non-heterosexual orientation; and currently resided in the United States.

### Study design

This survey was part of a larger study of tobacco messaging and tobacco use among SMW.^38^ Potential participants interested in the study reviewed a brief description about the study on Prolific. After providing consent, participants were directed to complete several questions, including measures of demographics, perceived stress, social support, and changes in health behaviors during the COVID-19 pandemic. On completing the survey, participants were compensated $3.17 via Prolific per Prolific's policies. All procedures were approved by The Ohio State University's institutional review board.

### Measures

#### Demographics

Participants reported their sexual orientation (defined as self-reported sexual identity and collapsed to bisexual, lesbian, and asexual/other), age, income (below $20,000, $20,000 to <$40,000, >$40,000), race, and ethnicity (collapsed to Non-Hispanic White, Non-Hispanic Black, Other/Multiple).

#### Perceived stress

Participants completed the 4-item perceived stress scale (PSS-4)^39^ assessing their frequency of feeling confident about handling personal problems and whether they felt that things were going the way they desired. Scores ranged from 1 (never) to 5 (very often) and were averaged (Cronbach's *α* = 0.77).

#### Social support

Social support was assessed with four questions about the frequency of receiving (1) emotional support from friends or loved ones, (2) material or financial support from friends or loved ones, (3) support from friends or loved ones through virtual platforms (*i.e.*, phone, FaceTime, Zoom, Skype, Facebook, WhatsApp or a similar app), and (4) in-person/face-to-face contact with friends or loved ones who do not live at home. Scores ranged from 1 (every day) to 5 (never, even though I wanted support) and 6 (I have not needed support) and collapsed to three levels: (1) every day or several times a week (*i.e.*, more than weekly), (2) weekly or monthly (*i.e.*, at least monthly), (3) never or not needing it (*i.e.*, never).

#### Changes in health behaviors during COVID-19 pandemic

The primary outcomes were changes in health behaviors during the COVID-19 pandemic. Participants were asked to indicate whether their (1) fruit and vegetable intake, (2) physical activity, (3) sleep, (4) combustible tobacco product use (*e.g.*, cigarettes, cigars, cigarillos, hookah), (5) e-cigarette or electronic nicotine product use (*e.g.*, refillable or disposable nicotine pods/vapes), (6) alcohol consumption, and (7) other substance use (*e.g.*, marijuana, stimulants, or other drugs) increased, decreased, or stayed the same as before COVID-19. Participants who responded that they never used a specific substance were excluded from respective analyses.

### Statistical analyses

We calculated descriptive statistics to report distributions of perceived stress, social support, changes in health and risk behaviors, and participant characteristics. We used multinomial logistic regression models to estimate associations of perceived stress and social support with changes in health behaviors during the COVID-19 pandemic. Models controlled for sexual orientation, age, income, and race and ethnicity. Separate models estimated the odds of increasing or decreasing each of the seven health behaviors compared with maintaining the same behavior as before COVID-19. Next, we estimated whether associations differed based on social support. In case of statistically significant interactions, we reported associations between perceived stress and changes in health behaviors by social support. Stata/SE version 17 was used for all analyses.

## Results

### Participants

On average, participants were 23.6 years old (standard deviation = 3.5) and primarily identified themselves as bisexual (76.5%), non-Hispanic White (60.3%) women with individual incomes below $20,000 per year (57.9%) (Table 1).

**Table 1.** Sample Characteristics, *N* = 501 Young Adult Sexual Minority Women, 2021

	*N* (%) or mean (SD)
Sexual orientation	
Bisexual	383 (76.4)
Lesbian	88 (17.6)
Asexual	26 (5.2)
Other	4 (0.8)
Race and ethnicity	
White (non-Hispanic)	301 (60.1)
Black (non-Hispanic)	50 (10.0)
Asian	56 (11.2)
Hispanic	36 (7.2)
Native American, Alaskan Native, Pacific Islander	2 (0.4)
Multiple races	54 (10.8)
Age (years)	23.6 (3.5)
Income	
<$20,000	290 (57.9%)
$20,000 to $40,000	105 (21.0%)
>$40,000	106 (21.2%)

SD, standard deviation.

### Associations between perceived stress and changes in health behaviors

#### Changes in fruits and vegetable consumption

A 1-U increase in the PSS was associated with higher odds of reporting decreased (odds ratio [OR] = 1.20, confidence interval [95% CI]: 1.06–1.26, *p* = 0.01) or increased fruit and vegetable intake (OR = 1.12, 95% CI: 1.00–1.26, *p* = 0.04), relative to maintaining pre-pandemic fruit and vegetable intake.

#### Changes in sleep

A 1-U increase in the PSS was associated with higher odds of reporting decreased (OR = 1.24, 95% CI: 1.08–1.41, *p* < 0.01) or increased sleep (OR = 1.20, 95% CI: 1.06–1.35, *p* < 0.01), relative to maintaining pre-pandemic sleep patterns.

#### Changes in alcohol, tobacco, and other substance use

A 1-U increase in the PSS was associated with higher odds of reporting increased other substance use (OR = 1.19, 95% CI: 1.01–1.39, *p* = 0.04), relative to maintaining pre-pandemic substance use behaviors. We found no statistically significant association between perceived stress and changes in combustible tobacco, e-cigarette, or alcohol use.

#### Changes in physical activity

Associations between perceived stress and changes in physical activity were not statistically significant.

### Associations between social support and changes in health behaviors

#### Changes in fruit and vegetable consumption

Virtual support was associated with changes in fruits and vegetable consumption. Compared with those who did not receive virtual support, participants who received virtual support at least monthly (OR = 0.18, 95% CI: 0.04–0.74, *p* = 0.02) or more than weekly (OR = 0.15, 95% CI: 0.04–0.62, *p* = 0.01) had lower odds of decreasing their fruit and vegetable intake, relative to maintaining pre-pandemic fruit and vegetable intake.

Those who received virtual support at least monthly (OR = 0.21, 95% CI: 0.05–0.83, *p* = 0.03) or more than weekly (OR = 0.16, 95% CI: 0.04–0.62, *p* = 0.01) had lower odds of increasing their fruit and vegetable intake, relative to maintaining pre-pandemic fruit and vegetable intake. Other forms of support were not associated with changes in fruit and vegetable consumption.

#### Changes in physical activity

In-person support was associated with physical activity. Compared with those who never received in-person support, participants who received in-person support more than weekly had lower odds of decreasing their physical activity (OR = 0.38, 95% CI: 0.16–0.91, *p* = 0.03), and those who received in-person support at least monthly had lower odds of increasing their physical activity (OR = 0.47, 95% CI: 0.22–1.00, *p* = 0.049), relative to maintaining pre-pandemic physical activity patterns. Other forms of support were not associated with changes in physical activity.

#### Changes in sleep

We detected no associations between social support and changes in sleep.

#### Changes in combustible tobacco use

In-person support was associated with changes in combustible tobacco use. Compared with those who never received in-person support, participants who received in-person support more than weekly had higher odds of decreasing combustible tobacco use (OR = 10.10, 95% CI: 2.79–36.58, *p* < 0.001) or increasing combustible tobacco use (OR = 7.35, 95% CI: 1.88–28.76, *p* < 0.01), relative to maintaining pre-pandemic tobacco use. Other forms of support were not associated with changes in combustible tobacco use.

#### Changes in e-cigarette use

In-person support was associated with changes in e-cigarette use. Compared with those who never received in-person support, participants who received in-person support at least monthly (OR = 6.64, 95% CI: 1.47–29.92, *p* = 0.01) or more than weekly (OR = 5.96, 95% CI: 1.12–31.77, *p* = 0.04) had higher odds of increasing e-cigarette use relative to maintaining pre-pandemic e-cigarette use. Other forms of support were not associated with changes in e-cigarette use.

#### Changes in alcohol use

Emotional support, material support, and in-person support were associated with changes in alcohol use. Compared with those who never received emotional support, participants who received emotional support more than weekly had lower odds of increasing their alcohol use (OR = 0.41, 95% CI: 0.18–0.94, *p* = 0.04), relative to maintaining pre-pandemic alcohol use.

Compared with those who never received material support, participants who received material support at least monthly (OR = 0.43, 95% CI: 0.21–0.88, *p* = 0.02) or more than weekly (OR = 0.32, 95% CI: 0.13–0.75, *p* = 0.01) had lower odds of increasing their alcohol use relative to maintaining pre-pandemic alcohol use. Compared with those who never received in-person support, participants who received in-person support more than weekly had higher odds of increasing their alcohol use (OR = 2.63, 95% CI: 1.23–5.60, *p* = 0.01) relative to maintaining pre-pandemic alcohol use. Virtual support was not associated with changes in alcohol use.

#### Changes in substance use

Material support was associated with changes in substance use. Compared with those who never received material support, participants who received material support more than weekly had lower odds of increasing their substance use (OR = 0.30, 95% CI: 0.10–0.95, *p* = 0.04) relative to maintaining pre-pandemic substance use. Other forms of social supports were not associated with changes in substance use (Table 2).

**Table 2.** Perceived Stress and Social Support on Health Behavior Changes During the COVID-19 Pandemic

	Fruits and vegetables			Physical activity			Sleep	
	Decreased		Increased	Decreased	Increased		Decreased	Increased
	OR (95% CI)		OR (95% CI)	OR (95% CI)	OR (95% CI)		OR (95% CI)	OR (95% CI)
Perceived stress	**1.20** (1.06–1.36)		**1.12** (1.00–1.26)	1.04 (0.92–1.18)	0.97 (0.83–1.12)		**1.24** (1.08–1.41)	**1.20** (1.06–1.35)
Emotional support								
Weekly/monthly	1.18 (0.50–2.80)		1.21 (0.54–2.71)	1.41 (0.59–3.33)	1.03 (0.37–2.88)		0.51 (0.20–1.3)	0.81 (0.33–2.02)
Everyday/several times a week	1.15 (0.49–2.67)		1.16 (0.53–2.55)	2.15 (0.91–5.07)	1.34 (0.48–3.71)		0.61 (0.24–1.52)	1.35 (0.55–3.33)
Material support								
Weekly/monthly	0.82 (0.41–1.66)		1.90 (0.86–4.21)	0.82 (0.33–1.99)	0.72 (0.25–2.11)		1.15 (0.49–2.71)	1.39 (0.64–3.04)
Everyday/several times a week	0.56 (0.23–1.34)		1.15 (0.44–2.95)	0.68 (0.23–2.03)	0.95 (0.27–3.38)		1.11 (0.40–3.09)	1.10 (0.43–2.79)
In-person support								
Weekly/monthly	1.00 (.56–1.77)		0.86 (0.52–1.42)	0.67 (0.34–1.30)	**0.47** (0.22–1.00)		1.09 (0.60–2.00)	0.97 (0.57–1.65)
Everyday/several times a week	1.32 (.56–3.09)		1.54 (0.74–3.20)	**0.38** (0.16–0.91)	0.53 (0.20–1.41)		0.90 (0.36–2.26)	1.30 (0.60–2.84)
Virtual support								
Weekly/monthly	**0.18** (0.04–0.74)		**0.21** (0.05–0.83)	0.75 (0.24–2.34)	2.38 (0.42–13.48)		0.46 (0.15–1.45)	1.31 (0.37–4.65)
Everyday/several times a week	**0.15** (0.04–0.62)		**0.16** (0.04–0.62)	1.53 (0.50–4.72)	2.63 (0.46–14.87)		0.68 (0.22–2.07)	2.27 (0.65–7.96)

	Combustible tobacco		E-cigarette		Alcohol		Substance	
	Decreased	Increased	Decreased	Increased	Decreased	Increased	Decreased	Increased
	OR (95% CI)	OR (95% CI)	OR (95% CI)	OR (95% CI)	OR (95% CI)	OR (95% CI)	OR (95% CI)	OR (95% CI)
Perceived stress	1.19 (0.99–1.42)	1.16 (0.94–1.44)	1.09 (0.87–1.36)	1.06 (0.86–1.32)	1.03 (0.91–1.17)	1.12 (1.00–1.26)	1.06 (0.85–1.32)	**1.19** (1.01–1.39)
Emotional support								
Weekly/monthly	0.48 (0.16–1.43)	0.63 (0.14–2.72)	0.33 (0.09–1.21)	1.36 (0.32–5.68)	0.46 (0.17–1.23)	0.43 (0.18–1.00)	0.46 (0.13–1.72)	0.84 (0.29–2.37)
Everyday/several times a week	0.64 (0.22–1.85)	1.03 (0.26–4.11)	0.43 (0.12–1.48)	1.13 (0.27–4.66)	0.51 (0.19–1.32)	**0.41** (0.18–0.94)	0.60 (0.17–2.07)	0.95 (0.34–2.63)
Material support								
Weekly/monthly	0.48 (0.19–1.25)	0.33 (0.11–1.01)	0.35 (0.12–1.04)	0.43 (0.16–1.17)	0.86 (.36–2.07)	**0.43** (0.21–0.88)	0.64 (0.21–1.98)	0.72 (0.31–1.70)
Everyday/several times a week	0.62 (0.15–2.57)	0.67 (0.14–3.15)	0.42 (0.08–2.25)	0.75 (0.18–3.05)	0.47 (0.16–1.38)	**0.32** (0.13–0.75)	0.51 (0.12–2.13)	**0.30** (0.10–0.95)
In-person support								
Weekly/monthly	2.63 (0.83–8.30)	1.94 (.59–6.40)	2.51 (0.88–7.16)	**6.64** (1.47–29.92)	1.38 (0.76–2.50)	1.36 (0.80–2.32)	1.06 (0.41–2.75)	0.95 (0.49–1.82)
Everyday/several times a week	**10.10** (2.79–36.58)	**7.35** (1.88–28.76)	2.28 (0.64–8.21)	**5.96** (1.12–31.77)	1.49 (0.59–3.74)	**2.63** (1.23–5.60)	2.18 (0.58–8.14)	1.77 (0.67–4.67)
Virtual support								
Weekly/monthly	0.94 (0.17–5.13)	0.49 (0.11–2.30)	—^a^	1.07 (0.18–6.40)	0.55 (0.16–1.92)	0.42 (0.14–1.30)	2.81 (0.30–26.28)	1.06 (0.30–3.78)
Everyday/several times a week	0.87 (0.16–4.64)	0.44 (0.10–1.96)	—^a^	0.70 (0.12–4.09)	0.41 (0.12–1.42)	0.48 (0.16–1.46)	1.65 (0.17–15.14)	0.72 (0.21–2.55)

OR are presented. Statistically significant results (*p* < 0.05) are denoted in bold. The models controlled for sexual orientation, age, income, race, and ethnicity. The reference group for the outcome variables stayed the same. The reference group for the independent variables was never or never needing support.

^a^ Results cannot be provided due to 0 participants in the reference group for virtual support decreasing their e-cigarette use.

CI, confidence interval; OR, odds ratios.

### Associations between perceived stress and social support on changes in health behaviors

The interaction of PSS and material support was significant on changes in alcohol use [*χ*^2^(4) = 15.29, *p* = 0.004]. Among participants who never received material support, a 1-U increase in PSS was associated with higher odds of increasing alcohol use (OR = 1.25, 95% CI: 1.07–1.47, *p* = 0.005), relative to maintaining pre-pandemic alcohol use. Association between PSS and changes in alcohol use was not significant among participants who received material support at least monthly and those who received material support more than weekly.

The interaction of PSS and material support was significant on changes in e-cigarette use [*χ*^2^(4) = 14.57, *p* = 0.006]. The *post hoc* tests were not statistically significant. Other associations were also not statistically significant.

## Discussion

Our findings highlight the associations of perceived stress and four types of social support with health behavior changes during the COVID-19 pandemic. Increased perceptions of stress were associated with changes in health (fruit and vegetable intake, sleep) and risk behaviors (substance use). Relatedly, social support was associated with changes in health (fruit and vegetable intake, physical activity) and risk (tobacco, alcohol, and substance use) behaviors. Further, increased perceived stress was associated with increased alcohol use among SMW who did not receive material support during the COVID-19 pandemic.

We identified associations between perceived stress and changes in health and risk behaviors. Increased perceptions of stress were associated with higher odds of both increased and decreased fruit and vegetable intake and sleep. These changes may indicate signs of eating and sleep disorders resulting from stress or healthy coping responses to overcome stress. In addition, increased perceptions of stress were associated with higher odds of increased substance use, which is consistent with prior research.^33,35,36^ Our findings underscore the need to address mental well-being among SMW to prevent the initiation of other substance use disorders and potential relapse of existing substance use.^36,40^

We also identified associations between social support and changes in health behaviors, including fruit and vegetable consumption and physical activity. Associations between virtual support and changes in fruit and vegetable intake were mixed, which indicates that virtual communications might support or impede healthy eating among SMW.

Associations between in-person support and changes in physical activity were also mixed, potentially suggesting that some in-person support might promote healthy behaviors, whereas others might not. For instance, frequent in-person contact with people who “sabotage” or undermine healthy behaviors may adversely impact the ability of sexual minoritized individuals to improve diet and physical activity.^41,42^

We also identified associations between in-person support and changes in tobacco use, including combustible tobacco and e-cigarettes. The association of in-person support and changes in combustible tobacco use was mixed in that receiving in-person support was associated with both increased and decreased combustible tobacco use. The mixed findings indicate that some in-person support may promote combustible tobacco use, whereas other in-person support can discourage SMW from use. Our results provide opportunities for future research to investigate the potential causes of these behaviors.

In addition, in-person support was associated with increased use of e-cigarettes, which may reflect social e-cigarette use evidenced among young people generally^43^ or LGBTQ-specific cultural norms that support social nicotine and tobacco product use.^28,29^

The association between social support and alcohol use was also mixed. For instance, receiving emotional support was associated with reduced alcohol use, possibly indicating that emotional support provided a buffer for SMW to positively cope with loneliness and psychological distress associated with COVID-19.^33,35,36^ Conversely, in-person support was associated with increased alcohol use, which potentially indicates that SMW used alcohol as a means to socialize or collectively cope.

Material support was associated with reduced alcohol and substance use, which potentially indicates that SMW lacked financial resources to procure alcohol or substance during the pandemic. However, increased perceived stress was associated with increased alcohol use among SMW, who reported never receiving material support. It is possible that a lack of material support limits financial accessibility to alcohol, yet experiencing financial challenges exacerbates stress among SMW, triggering greater use of alcohol to cope.

Overall, our findings indicate that social support can positively or negatively influence SMW to change their health and risk behaviors. Specifically, associations between social support and increased risk behaviors (*i.e.*, tobacco, e-cigarette, alcohol, and substance use) indicate a need to explore diverse avenues to appropriately increase social support for SMW.^42^

### Limitations and future directions

Our study has several limitations. First, we used convenience sampling and, thus, results cannot be generalized to all SMW in the United States. Second, data were collected using self-report measures, which has a potential for social-desirability bias and recall bias associated with reporting pre-pandemic behaviors that would have occurred over 10 months before the study. In addition, some of our findings are imprecise due to small cells. Finally, although we could speculate based on the literature, we could not determine the reasons for mixed findings. Future studies may involve additional questionnaires or qualitative investigations to identify the types of social support that are helpful or unhelpful.

Our findings also present opportunities for future research. Given that 60% of the sample consisted of Non-Hispanic White women, results might differ in a sample with greater racial and gender diversity (*e.g.*, SMW of color, gender minoritized women) who experience greater social inequities from COVID-19 than their White or cisgender SMW counterparts.^4^ Future studies may purposely oversample racial and ethnic minoritized women to explore the intersections of race and sexual orientation, and how social support can buffer the compounded effect of stress related to both COVID-19 and marginalized social status to improve healthy behaviors.

## Conclusion

This study underscores the importance of perceived stress and social support on health and risk behaviors among SMW. To our knowledge, this is the first study to examine how stress and social support were associated with changes in leading health indicators among SMW during the COVID-19 pandemic. Before the COVID-19 pandemic, SMW evidenced disparities in several health and risk behaviors, including nutrition, alcohol, tobacco, and substance use.^5–8,33,34^

Our findings indicate that perceived stress is associated with changes in fruit and vegetable intake, sleep, and substance use whereas various types of social support are associated with both positive and negative changes in fruit and vegetable intake, physical activity, combustible tobacco and e-cigarette use, alcohol, and substance use. These findings provide preliminary evidence that the COVID-19 pandemic may have exacerbated pre-pandemic health disparities in this population, through increased stress and unhelpful social support. Finally, results also suggest that existing social support systems might be insufficient to help SMW engage in healthy behaviors, and there is a need to bolster buffers against stress for this population.

## Abbreviations Used

CI: confidence interval
LGBTQ: lesbian, gay, bisexual, transgender, and queer
OR: odds ratio
PSS-4: perceived stress scale
SD: standard deviation
SMW: sexual minoritized women
